# The Role of Posttranslational Protein Modifications in Rheumatological Diseases: Focus on Rheumatoid Arthritis

**DOI:** 10.1155/2015/712490

**Published:** 2015-05-18

**Authors:** Andrea Mastrangelo, Tania Colasanti, Cristiana Barbati, Arbi Pecani, Danilo Sabatinelli, Monica Pendolino, Simona Truglia, Laura Massaro, Riccardo Mancini, Francesca Miranda, Francesca Romana Spinelli, Fabrizio Conti, Cristiano Alessandri

**Affiliations:** Reumatologia, Dipartimento di Medicina Interna e Specialità Mediche, Sapienza Università di Roma, Italy

## Abstract

The definition of posttranslational modification (PTM) encompasses a wide group of chemical reactions that allow modification and modulation of protein functions. The regulation of PTMs is crucial for the activity and survival of the cells. Dysregulation of PTMs has been observed in several pathological conditions, including rheumatoid arthritis (RA). RA is a systemic autoimmune disease primarily targeting the joints. The three PTMs mainly involved in this disease are glycosylation, citrullination, and carbamylation. Glycosylation is essential for antigen processing and presentation and can modulate immunoglobulin activity. Citrullination of self-antigens is strongly associated with RA, as demonstrated by the presence of antibodies directed to anti-citrullinated proteins in patients' sera. Carbamylation and its dysregulation have been recently associated with RA. Aim of this review is to illustrate the most significant alterations of these PTMs in RA and to evaluate their possible involvement in the pathogenesis of the disease.

## 1. Introduction

Human cells are able to maintain high levels of efficiency and organization thanks to a complex and finely regulated network of numerous processes (e.g., DNA transcription, protein synthesis). The proper managing of these processes strictly depends on a large group of chemical reactions named posttranslational modifications (PTMs). A PTM consists in every change of chemical structure or property of a protein that occurs after or at the same time of its translation. Nowadays, over 300 PTMs are known [1] and over 200 of them are enzyme-mediated [2], highlighting the attempt of the cell to exploit these modifications for surviving. PTMs are crucial for the development and evolution of every living organism, and it is universally accepted that the more the species are advanced, the better they use and organize PTMs [3]. PTMs and their dysregulations in pathological conditions gained great interest, due to the continuous improvement in biotechnologies that allow better investigation of and definition of these processes.

Rheumatoid arthritis (RA) is an autoimmune disease affecting about 1% of the general population and it is characterized by polyarticular, symmetric involvement of synovial joints, as well as several extra-articular manifestations, such as rheumatoid nodes, pulmonary fibrosis, and accelerated atherosclerosis [4]. The pathogenesis of RA has not been completely elucidated yet and although in these last decades the use of new therapeutic agents has improved the prognosis of the disease, RA is still an important cause of morbidity and disability.

The aim of this review is to give an overview on the role of PTMs in the pathogenesis of RA, focusing on the modifications that have been strongly associated with the disease: glycosylation, citrullination, and carbamylation (Table 1).

## 2. Glycosylation in Rheumatoid Arthritis

**Figure 1:**
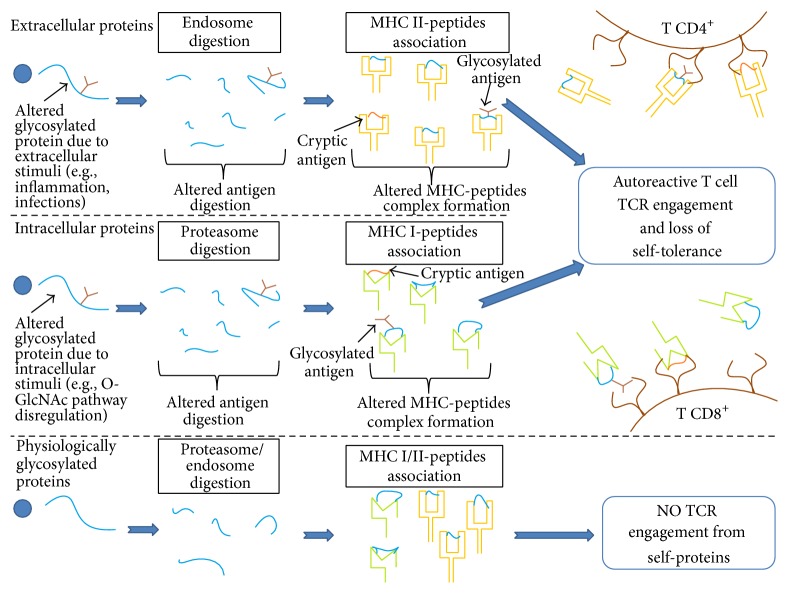
Aberrant glycosylation affects antigen presentation and can induce breaking of tolerance. (Of note, due to the cross-presentation pathway, intracellular proteins with altered glycosylation could be presented on MHC II molecules to T CD4^+^).

Glycosylation consists in the addition of sugars on nitrogen (N-glycosylation) or oxygen (O-glycosylation) atoms of the side chain of the protein amino acids. This reaction is mediated by hundreds of glycosyl-transferases (GTs), regulated basically at transcriptional level. Human cells modify simple sugars, such as glucose or galactose, in more complex ones, in order to create a larger number of precursors for glycosylation; subsequently, the linking of uridine diphosphate or, rarely, cytosine monophosphate activates the sugars. Glycosylation occurs on both extracellular and intracellular proteins and it is crucial for several vital processes such as proteins folding or cell-to-cell interactions [5]. Glycosylation is involved in two physiopathological processes—epitope presentation to immune system and immunoglobulins- (Igs-) mediated regulation of immune response—suggesting a possible association of aberrant glycosylation with RA (Figure 1). Furthermore, aberrant glycosylation of different plasma proteins was demonstrated in RA patients.

### 2.1. Glycosylation and Epitope Presentation

Glycosylation affects the antigen presentation acting both on antigen processing pathways in antigen presenting cells (APCs) and on T cell receptor- (TCR-) MHC II complex formation.

Glycosylation plays an important role in antigen presentation by modifying the cleavage of proteins in both proteasome (MHC I-associated pathway) and endosome (MHC II-associated pathway). A considerable number of cytosolic peptides presented on MHC I molecules carry an O-glycosylation with a molecule of N-acetyl-glucosamine (GlcNAc) [6]. The O-GlcNAcylation is a PTM that interacts with phosphorylation [7] to regulate several intracellular processes, such as the activation of nuclear transcription factors and the responses to nutrient deprivation. Abolishing or modifying the physiological O-GlcNAcylation processes, as observed during inflammation or other responses to stressful stimuli [8], could determine the presentation of new antigenic glycopeptides on MHC I molecules that can bind to autoreactive T cells.

Regarding MHC II-associated pathway, it was demonstrated that different glycoforms of the same protein are processed in different ways, generating different peptides linked on MHC II molecules; this process can lead to the presentation of cryptic self-antigens or to an enhanced presentation of normally nonimmunodominant self-antigens [9].

During the antigen presentation, APCs are not capable of cleaving the side sugar chains; so, if a certain peptide carries a glycan side chain, the whole glycan-peptide complex will be presented on MHC II molecule. This process may improve or diminish TCR affinity for the MHC-peptide complex by steric interference [10].

Glycosylation influences T lymphocyte activation. Many studies investigated T cell responses to subtypes of collagen II-derived peptides in mice with collagen induced arthritis (CIA) [11, 12]. Corthay and coworkers pointed out that the response to these peptides is strictly dependent on the PTM carried on lysine 264 (K264). B10.Q mice showed a strong response to galactosylated K264 and a very weak response to unmodified or hydroxylated K264 collagen derived peptides [13]. As expected, mice immunization with galactosylated K264/MHC II complex reduced the severity of arthritis and the extent of the humoral [anti-type II collagen (anti-CII) antibodies] immune response [14]. Recently, Batsalova et al. demonstrated that DR4 transgenic mice (expressing human HLA-DR4 allele) are more responsive to the unmodified or hydroxylated K264 compared to the galactosylated one [15]. These results are in accordance with the previous observation that collagen from human and rats with active arthritis contains glycosylated and unglycosylated zones; on the contrary, in healthy subjects collagen is uniformly glycosylated [16]. Besides collagen, other proteins—for example, p68—show a glycosylation-dependent T cell recognition [17]; hence, an aberrant glycosylation might be one of the triggers for the onset and progression of RA.

Infections as well lead to an alteration of self-protein glycosylation.* E. coli* is able to modify glycosylation of self-proteins, due to its own GTs expression [18].* H. pylori* CagA toxin could also alter the glycosylation processes in B lymphocytes [19]. Moreover, the cytokine secretion induced by these (and other) pathogens is able to modify the cellular pattern of GTs [20, 21]. All together, these events could be a rational explanation for the theory of “infection-triggered autoimmunity.”

### 2.2. Glycosylation and Immunoglobulin Properties

As previously mentioned, glycosylation may affect the immune system also by modifying IgG properties. Fc fragment of IgGs has an important site of N-glycosylation, the 297 asparagine (N297). IgGs can exert both proinflammatory and anti-inflammatory activities depending on which Fc*γ* receptor they preferentially bind: the different receptor affinity strictly depends on the composition of the sugar side chain linked to N297 [22, 23]. The phenotype associated with a higher proinflammatory activity displays low levels of galactose and sialic acid [24]; differently, the anti-inflammatory phenotype is characterized by normal galactose and sialic acid and reduced GlcNAc levels [25, 26]. IgGs can exert their anti-inflammatory effects also by binding to other receptors: whether this interaction is dependent or not on Fc fragment glycosylation is still unclear [27–29]. Different factors, such as interleukins or lipopolysaccharide, can induce a proinflammatory pattern of the sugars linked to IgGs [30, 31]. This whole process finally leads to a vicious circle of self-sustaining immune activation.

In active RA, anti-citrullinated peptide antibodies (ACPA) and rheumatoid factor (RF) display the proinflammatory N297 glycosylation pattern, with low levels of galactose and sialic acid [32–34]. These autoantibodies acquire this glycosylation pattern before the clinical onset of the disease [35]; interestingly, changes in IgGs glycosylation were associated with RA remission observed during pregnancy [36, 37]. Taken together, these observations remark the crucial role of IgG glycosylation in the pathogenesis of the disease.

Many circulating proteins can also display an altered glycosylation in RA [38–40]. For example, lubricin isolated by synovial fluid of RA patients expresses an aberrant glycan determinant and shows an L-selectin ligand activity that may induce the activation of neutrophils and polymorphonuclear cells [40].

A good response to therapy turns the IgGs glycosylation back to a noninflammatory phenotype [41]. In order to stop the vicious circle triggered by the altered IgGs glycosylation, Nandakumar et al. proposed a treatment with endostreptosin (EndoS)—a bacteric glycosidase able to trim the whole N297 side chain.* In vitro* incubation of anti-CII monoclonal antibodies with EndoS prevents the onset of arthritis in mice; this effect persists even if antibodies-EndoS are injected together with anti-CII antibodies [42].

Some authors hypothesized a possible treatment of severe RA and, in general, of autoimmune diseases with sialic acid enriched-intravenous Igs; however, the preliminary results are not conclusive and call for further researches [43, 44].

## 3. Citrullination and Rheumatoid Arthritis

**Figure 2:**
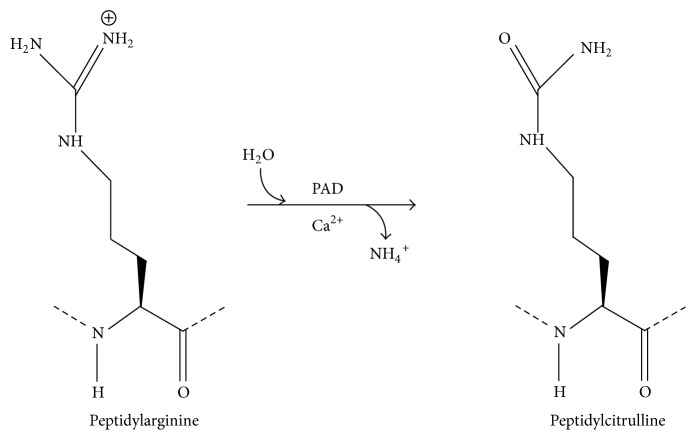
Biochemical process that occurs in protein citrullination event.

Citrullination is a protein modification consisting in the switch of the iminic nitrogen of arginine to oxygen, linked to the backbone structure as a ketone; this process results in the production of an amino acid called citrulline. The reaction is mediated by a family of enzymes called peptidylarginine deiminase (PAD or PADI) [45] (Figure 2). Nowadays, 5 subtypes of PAD (PAD1-6), variously distributed in human cells, are known [46, 47]. The activity of these enzymes is regulated by intracellular Ca^2+^ concentration. The subtypes of PAD expressed by the immune cells are PAD4 and PAD2, markedly present in neutrophils and mast cells [48]. The switch from arginine to citrulline has several different consequences on protein structure: the oxygen of citrulline is a noncharged atom, differently from the positive-charged nitrogen of arginine; since side chain charge of the amino acid sequence is crucial for the protein folding, an alteration in the electronic milieu modifies the tertiary structure of the peptide [49]. So far, citrullination is associated with histone modifications, neutrophils extracellular trap (NET) formation, and epidermal, central nervous system and skeletal muscle tropism regulation [47].

Immunogenicity of citrullinated proteins has been studied, especially in autoimmune diseases such as RA, leading to the identification of ACPA. Nowadays, ACPA are essential in RA diagnosis: the presence of these autoantibodies, revealed with the ACPA assays, has a specificity of 85–95% and a sensitivity of about 80% [50].

ACPA can be identified in about 50% of RA patients approximately 1 year before the onset of arthritis [51]. Higher titre of ACPA at diagnosis is considered a negative prognostic factor and ACPA titre seems to be reduced after treatment [52, 53]. Furthermore, citrullinated antigens recognized by ACPA are abundant in inflamed synovia; among the others, alpha-1 antitrypsin, fibrinogen, apolipoproteins, histones, immunoglobulins, and vimentin had been characterized [54].

All these findings depose even for a pathological role of these autoantibodies in the development of RA.

Two questions arising from these data still remain partially unsolved: why do RA patient antibodies recognize as “non-self” the citrullinated proteins? And how citrullinated antigens are produced?

The first question found a partial answer in the strong association between ACPA-positive RA and specific HLA polymorphisms, especially the conserved region of HLA-DRB1^*^0104 allele: subjects who carry this allele have an higher risk of developing an erosive ACPA-positive RA, due to the ability of the HLA molecule coded by this specific haplotype to bind and present citrullinated antigens [55]. Nevertheless, this haplotype is also diffused in healthy population. Other susceptibility genes have been identified, but altogether they represent only 50% of genetic variance associated with ACPA-positive arthritis [56]. Clearly, more studies are needed to elucidate the physiopathological pathway leading to ACPA formation.

The latter question is much more complex. In fact, even if the final step of citrullination always consists in the activation of the PAD-family enzymes, the modulation of this pathway is variable and still not completely understood.

Summarizing, the processes leading to the formation of citrullinated epitopes can result in an accumulation of citrullinated proteins at extracellular and/or intracellular levels, which will be subsequentely described.

### 3.1. Extracellular Accumulation

Extracellular accumulation of citrullinated proteins is related to two main factors: NET formation and* P. gingivalis* infection. NETosis is a powerful defensive mechanism developed by immune system in order to trap and destroy extracellular bacteria. NETs are constituted by loose chromatin complexed with many antimicrobial enzymes stored in neutrophils granules; this whole complex is expelled by the cell in the extracellular space. The negative charge of chromatin traps the bacteria, allowing the enzymes to kill the microorganisms [57]. To undergo NETosis, neutrophils need PAD4 activation: the histone citrullination mediated by this enzyme causes chromatin relaxation that is crucial for the subsequent processes [58]. NETosis has been associated with several pathological conditions, like thrombosis, multiple sclerosis, and RA [59]. In RA, several alterations in NET formation have been noticed. When compared to healthy subjects, neutrophils isolated by the peripheral blood of RA patients undergo NETosis more easily; NETosis can be induced by RF or ACPA and, finally, the enzyme load of the chromatin shows differences between NETs of RA patients and healthy subjects [60].

In an inflammatory contest, such as the rheumatoid synovia, NETs release citrullinated antigens [61]: in RA predisposed individuals, synovial citrullinated antigens may be caught and presented to T lymphocytes, promoting a local immune response.

*P. gingivalis*, a Gram negative, nonmotile, anaerobic bacterium, is considered the major causative agent of periodontitis in humans [62]. Periodontitis is an inflammation of the structures surrounding the teeth, affecting about 50% of the adult population [63]. It is also a well-studied risk factor for the development of RA [64], since* P. gingivalis* is the only bacterium able to synthesize its own PAD [65]. This bacterium can lead to an increase in citrullination of host periodontium proteins, creating new citrullinated epitopes [66].

Arandjelovic et al. hypothesized a new pathway for extracellular citrullination:* in vitro*, ATP from dying cells can bind an ATP-receptor on mast cells and promote the activation and the release in the extracellular space of PAD2 [67].

### 3.2. Intracellular Accumulation

Several mechanisms of intracellular accumulation of citrullinated proteins have been demonstrated* in vitro* and they represent an interesting field of research.

Autophagy is a cellular process consisting in the fusion of lysosomal vacuoles with vacuoles coming from intracellular elements, in order to digest them and restore energetic and nutrient reserve. Autophagy plays an important role in the regulation of intracellular organelle turnover [68]. Physiologically, this process occurs in starvation or under other stressful conditions for the cell and it has been also associated with several pathological conditions [69], including RA. During amino acid deprivation in B lymphocyte cell cultures, the induction of autophagy pathways leads to the intracellular activation of PAD and to the citrullination of self-peptides occurring during the MHC-peptide complex formation; moreover, treatment of B lymphocytes with both anti-IgG and anti-IgM results in an enhanced presentation of citrullinated antigens, abrogated by the addition of autophagy inhibitors;* in vivo*, the same mechanism may be mediated by RF [70].

Romero et al. demonstrated that membrane disruption can cause intracellular citrullination. This group showed that neutrophils treated with granzyme/perforin B or complement membrane attack complex undergo necrosis; the loss of membrane integrity led to a massive influx of Ca^2+^ and to an uncontrolled activation of PAD enzymes. The so-called hypercitrullination deriving from these events produces an elevated number of citrullinated proteins. Therefore, it can be hypothesized that* in vivo* an immune response directed to neutrophils could result in the formation of self-citrullinated antigens with a subsequent breaking of tolerance [71].

### 3.3. Smoking and Citrullination

Smoking is a risk factor for the development of ACPA-positive RA, especially in people who present HLA-DRB∗0104 allele [72]. This habit can increase the formation of citrullinated proteins and promote an activation of the immune system. Nanoparticles inbreathed with tobacco smoke can lead to the activation of PAD2 and PAD4 inside the cells [73]. Smoke effects on the cells include upregulation of PAD2 expression and subsequent citrullination [74].

To point out the crucial role of other predisposing factors in the onset of RA, Bongartz et al. revealed that lung biopsies of smokers that have developed lung cancer did not show any intracellular protein citrullination, in contrast with the strong intracellular protein citrullination detected in RA affected smokers [75].

### 3.4. PAD Inhibitors

Recently, several authors studied inhibitors of PAD for the treatment of RA. Cl-amidine, a pan-PAD inhibitor, showed an efficacy in mice with CIA in reducing severity of disease, synovial citrullination, and histological joint damage [76]. Another PAD inhibitor, the compound named YW3-56, showed a 5-fold increase in PAD4 activity inhibition, if compared with Cl-amidine. YW3-56 has been tested as anticancer drug, but its ability in blocking PAD4 could make it a good candidate also for the treatment of RA [77].

## 4. Carbamylation and Rheumatoid Arthritis

Carbamylation is a nonenzymatic PTM that consists in the addition of a cyanate group (O=C=N^−^) on self-proteins [78]. This reaction usually affects the atoms of nitrogen, although a certain degree of carbamylation has also been demonstrated on sulphur atoms [79]. Free nitrogen atoms in proteins can be found at the N-terminus or in the side chain of lysine and arginine. The most important carbamylation detected in patient with RA is the lysine carbamylation: this chemical process leads to the formation of homocitrulline, a noncanonical amino acid, through the link of the carbon atom of cyanate with the nitrogen atom of the lysine [80].

Cyanate required for carbamylation can be produced in two different ways.

The first way is the spontaneous degradation of blood urea. In fact, urea is in equilibrium with cyanate [81], and the equilibrium in cyanate-urea ratio* in vivo* seems to oscillates around 1 : 200 [82].

The second way consists in cyanate intake from the external environment. Cyanate is found at a concentration of about 200 parts-per-trillion by volume in urban air and can be detected in both tobacco and biomass smoke [83]; potassium and sodium cyanate are used in several herbicides [84]; finally, many foods (e.g., broccoli) possess a moderate quantity of thiocyanate that, reacting with reactive oxygen species, can be converted in cyanate [85, 86].

In healthy individuals, the concentration of blood cyanate is about 50 nmol/L, an amount that is 1000 times lower than the expected one, according to urea kinetic equilibrium [87]. For some authors, this alteration can be explained with a sort of “physiological carbamylation” of N-terminus of blood proteins [88].

Carbamylation is time dependent: structural proteins, due to their slow turnover, are indeed much more likely to be carbamylated, if compared to proteins with shorter half-life [89].

**Figure 3:**
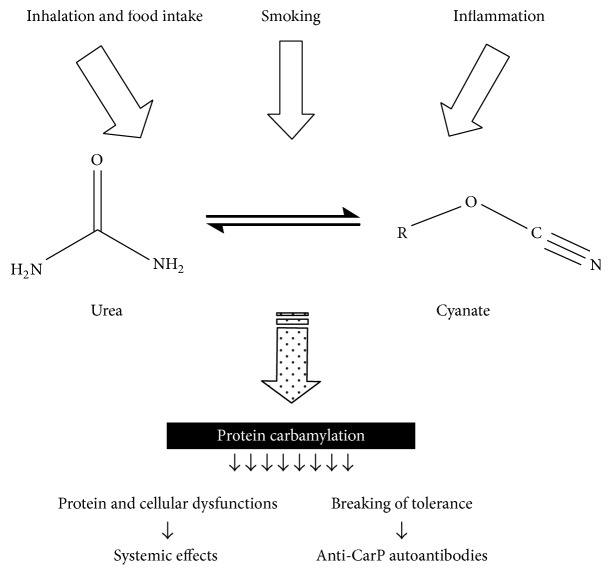
Protein carbamylation: causes and consequences. Several conditions can shift the balance between urea and cyanate towards cyanate production, enhancing in this way the process of protein carbamylation. This event results in systemic effects and production of anti-CarP autoantibodies.

Carbamylation of self-proteins causes a loss of the native proteic structure and can lead to a break of tolerance, finally resulting in the formation of anti-carbamylated proteins (anti-CarP) autoantibodies (Figure 3). These autoantibodies can be detected in RA patients. Inhibition assays demonstrated that ACPA and anti-CarP are 2 different families of autoantibodies [90].

Immunization with homocitrullinated peptides can cause T and B cell mediated immune response against the synovial membrane, and the intrarticular injection of homocitrulline-containing peptides can result in the development of a mild arthritis even in nonimmunized mice. Interestingly, in the study of Mydel and coworkers it was demonstrated that mice immunization with homocitrulline followed by the intrarticular injection of citrullinated or homocitrullinated peptides determined a severe arthritis in a higher percentage of the first group (more than 90%), suggesting this an unexpected response as a final linkage between alteration in citrullination and severe arthritis development [91].

Anti-CarP autoantibodies can be detected in about 16% of seronegative RA patients; their presence is correlated to a more severe and erosive disease [90] and represents a risk factor for developing RA in patients with inflammatory arthralgia [92]. Anti-CarP autoantibodies have been detected in RA patients' sera before the clinical manifestation of disease [93]. So, they appear to be a new useful tool in the diagnosis and follow-up of RA.

## 5. Conclusion

PTMs are certainly implicated in the development of RA and in several other autoimmune and nonautoimmune diseases. Nowadays, many aspects of RA pathogenesis have been understood, thanks to the large number of studies focusing on the role of PTMs in the induction of this disease. However, none of the theories proposed to explain the exact aetiology of RA is completely exhaustive. Every single PTM alteration alone is not sufficient to ignite the disease, so overlap of multiple alterations seems to be needed for the onset of RA in predisposed patients.

The challenge for the future studies is to find a linkage between the various PTM alterations that occur during the development of RA, in order to identify possible steps leading to the breaking of self-tolerance and to the clinical onset of the disease. Improving knowledge in the pathogenesis of RA could offer new suggestions for the development of more effective drugs.

## Figures and Tables

**Table 1:** Summary of some posttranslational modifications in rheumatoid arthritis.

PTM	Effect(s)	Reference

**Glycosylation**: PTM that consists in the addiction of glucides on an atom of nitrogen (N-glycosylation) or oxygen (O-glycosylation) of the lateral chain of the amino acids that forms a protein.	**Epitopes presentation**: -Protein glycosylation interacts with the processes of antigen presentation. -These interactions regard both antigen processing pathways in APCs and TCR-MHC II complex formation.	Haurum et al., 1999 [6] Jefferis et al., 1995 [22]
	**Igs properties**: -Fc fragment of immunoglobulins has an important site of N-glycosylation, the asparagine 297 (Asn297). -It has been shown that IgG can have both proinflammatory and anti-inflammatory activity, depending on which Fc*γ* receptor they preferentially bind to: those different affinities for receptors are strictly dependent on the composition of the saccharine lateral chain linked to Asn297.	Goulabchand et al., 2014 [23]

**Citrullination**: PTM consisting in the switch of the imine nitrogen of an arginine to an atom of oxygen, linked to the backbone structure as a ketone. It is mediated by a family of enzymes called PAD (or PADI).	-It is associated with histone modification, genomic regulation and NET formation. -PAD activation can be an intracellular or extracellular event, due to various conditions. -This activation can lead to the creation of altered self-epitopes and ACPA formation.	Yamada et al., 2005 [49] Khandpur et al., 2013 [60]

**Carbamylation**: Nonenzymatic PTM that consists in the addiction of a cyanate group on proteins.	-Loss of the native proteic structure. This event can lead to a break of tolerance and finally results in the formation of anti-CarP autoantibodies.	Shi et al., 2011 [90]

ACPA: anti-citrullinated protein antibodies; anti-CarP: anti-carbamylated protein; APC: antigen presenting cell; NET: neutrophil extracellular trap; PAD: peptidylarginine deiminase; PTM: posttranslational modification.
